# Associations between dental caries and ponderal growth in children: A Cambodian study

**DOI:** 10.7189/jogh.12.04046

**Published:** 2022-06-18

**Authors:** Bathsheba Turton, Tepirou Chher, Sithan Hak, Karen Sokal-Gutierrez, Diego Lopez Peralta, Arnaud Laillou, Ankur Singh

**Affiliations:** 1 University of Puthisastra, Phnom Penh, Cambodia; 2 Henry M. Goldman School of Dental Medicine, Boston University, Boston, Massachusetts, USA; 3 Oral Health Bureau, Department of Preventive Medicine, Ministry of Health, Phnom Penh, Cambodia; 4 University of California Berkely, California, USA; 5 Centre for Epidemiology and Biostatistics and Melbourne Dental School, University of Melbourne, Melbourne, Australia; 6 UNICEF Cambodia, Phnom Penh, Cambodia

## Abstract

**Background:**

The evidence around the relationship between Early Childhood Caries (ECC) and undernutrition is sparse and mostly reported from cross-sectional data sets. This paper aimed to test the relationship between ECC and linear and ponderal growth trajectories.

**Methods:**

This project involves secondary data analysis from the Cambodia Longitudinal Health and Nutrition Study. The analytical sample included a 2y-cohort of 894 children who were younger than 2 years of age at the time of first height and weight measurement. Statistical analysis used both logistic regression modelling and Latent Class Analysis to examine the effect of exposure to dental caries in the first 1000 days on weight for height Z-score (WHZ) and height for age Z-score (HAZ) trajectory class groups. The presence of any cavity and pulp involvement were examined using multinomial regression adjusting for gender, socioeconomic status, maternal age and education.

**Findings:**

Within each class groupings (HAZ and WHZ groupings), there was a trend whereby those with one or more cavities had lower Z-scores across the three follow-up time points of observation. There was an association between exposure to caries and WHZ class membership whereby children with caries exposure were more likely belong to WHZ class groups with lower Z-scores over time.

**Conclusions:**

The study offers evidence that ECC is correlated with less favourable ponderal growth categorized by WHZ trajectory class groups.

If (ECC) were to be a modifiable cause of sub-optimal growth and development, then this would be of particular interest in a context like Cambodia where there is a high prevalence of stunting and wasting undernutrition. Caries is a progressive disease mediated by bacteria and driven by dietary free sugars [1]. Once a caries lesion is cavitated, then the damage is irreversible and the lesion will advance to the vital structures of the tooth in the pulp space leading to pain among other symptoms. In Cambodia 36% of 4-year-olds are stunted [2], and 86% of children have had a dental infection by the age of six [3]. In 2015 Cambodia qualified as a ‘lower middle income country’ after sustained growth of 7.6% in the preceding decade [4]. Subsequently the proportions of children who were undernourished has reduced dramatically, however, the high prevalence of stunting remains persistent even ahead of the COVID-19 pandemic [2].

A recent systematic review concluded that the balance of evidence supports a hypothesis that ECC is associated with higher rates of stunting and wasting. However, the quality of the available evidence in most investigations was limited, and of cross-sectional design (33 of 38 included studies) [5], which could not establish causality. The relationship between ECC and undernutrition is complex as both conditions are influenced by the social determinants of health. The biological mechanisms for a relationship between ECC and growth and development is thought to be mediated through chronic infection, pain, loss of sleep, loss of function from caries-related tooth destruction and loss of appetite [6,7].

A typical way to examine undernutrition is to use the stunting or wasting outcome at a single time point and this poses a problem for some populations or subpopulations do not conform to normal growth standards. The stunting and wasting categories are defined by the respective height for age or weight for height Z-scores falling below 2 standard-deviations from ‘normal’ and in that way identify children who may be experiencing impacts of undernourishment. As such, it fails to take into account those individuals who may have slightly higher scores (just above -2) but who would also be likely to be experiencing the negative impacts of undernutrition. While these outcome measures have served well to offer an overview comparison among certain populations in broad terms, where there is a sub-population who are skewed towards negative z-score values, then those same measures may not capture clinically relevant variation within the group. This study uses growth trajectory class groupings defined through Latent Class Analyses (LCA) as the outcome of interest. This helps to test the hypothesis that ECC is associated with less favourable linear and ponderal growth trajectories among a specific sub-population of Cambodian children.

## METHODS

### Study settings and data source

This was a secondary analysis examining longitudinal data on health, nutrition, and diet of pregnant and lactating women along with their preschool children (<5 years of age), collected across two of three provinces included in the Cambodia Health and Nutrition Monitoring Study (CAHENMS). This investigation was a collaborative study among three organizations; the Ministry of Health, UNICEF Cambodia, and the French National Institute for Sustainable Development (IRD) to provide enhanced monitoring of health and nutrition in six districts of Cambodia. The Oral Health Bureau, Ministry of Health Cambodia added an oral health module at the fourth follow-up point after securing funding from The Borrow Foundation. The Study provided a unique opportunity to observe nutritional status and health indicators such as diarrhoea and acute respiratory infections. Several reports have been published which examine diet and feeding practice, epidemiological measurement of growth and development and contributors to stunting and wasting in a Cambodian environment [8-10].

Data from participants in Ratanakiri and Kratie province, only two of the three provinces in the broader study, were included in this analysis as funding limitations did not allow for data to be collected in Phnom Penh at the most recent data collection time point (May 2019). Ratanakiri is the most northeastern province of Cambodia, the population density is low and villages are scattered. Kratie is another of the northeastern provinces and it borders Vietnam to the South. Data were collected from the two central administrative districts of the province (Chet Borei and Krong Kratie). Kratie province displays two distinct regions whereby the northeast part of the province is a forested plateau suitable for cattle rearing and plantations. The Southwest is a wet plain fertilized by the Mekong River, where smallholder farmers successfully grow rice, corn, bean and horticulture products. In both agro-ecological regions, large amounts of land have been turned to Economic Concession zones.

These provinces were identified by UNICEF for Enhanced Health Monitoring over a 3-year period from 2016. The protocol for the study was reviewed by the National Ethics Committee for Health Research, Ministry of Health Cambodia (117/NECHR) and the protocol for the secondary data analysis was reviewed by the Research Committee at the University of Puthisastra. Participants gave written consent upon entry into the study and then verbally at each follow-up. Participants were followed across six follow-ups (FUP1- 6), finishing in May 2019; this analysis used data from FUP3 through to FUP6. Questionnaires, in Khmer language, were based on the demographic health survey question set to elucidate variables related to sociodemographic characteristics [2]. Trained native Khmer speakers administered questionnaires to the caregiver of each participating child as described in previous publications [11].

Oral examinations were conducted by calibrated senior dental students with the child in a supine position, using a mouth mirror and illumination from a handheld torch. Data were collected on caries status using the three stage ECC index and the Pulpally involved Ulcerated Fistula Abscess index [12] as described in previous publications [11].

### Exposure

The exposure was the presented in an ordinal scale based on ECC experience at FU4. The categories were: no cavitated caries lesions present; one or more cavitated caries lesions present; or one or more carious teeth with pulpal-involvement.

### Covariates

**Figure 1:**
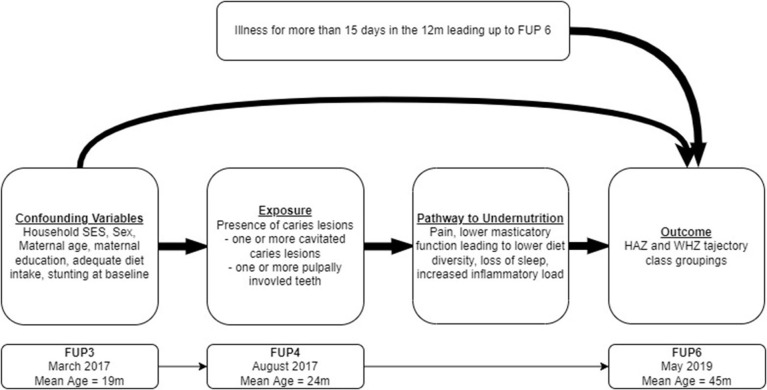
Timeline and directed acyclic graph for relationship between ECC and stunting SES – socio-economic status; HAZ – height-for-age Z-score; WHZ – weight-for-height Z-score; FUP – follow-up.

A minimal and guaranteed set of covariates were selected based on the literature. Common causes of ECC and stunting or wasting undernutrition (sex, location, household SES, maternal age, maternal education) were accounted for as confounding factors. Covariates were included at FUP3 (prior to measurement of ECC) in the 12 months prior to FUP3 to maintain temporal ordering. The presumed theoretical relationship between ECC and stunting is presented in a Directed Acyclic Graph (DAG) in **Figure 1****.**

### Outcome

LCA was used to explore the heterogeneity and determine classes of WHZ and HAZ trajectories from FUP4 to FUP6 [13]. This analysis assumed that all associations between the observed variables were due to unobserved classes. [14,15] LCA accounted for correlations between repeated measures of WHZ and HAZ scores for each child when defining the classes. [14-16]. The subjects assigned to each class were like each other according to the descriptor variables used, and the latent classes should correspond to clusters of similar subjects. Two sets of variables were estimated: conditional probabilities (ie, WHZ or HAZ scores at each time point within a known class) and posterior probabilities (ie, probability of membership of each WHZ or HAZ class).

### Analytical sample

**Figure 2:**
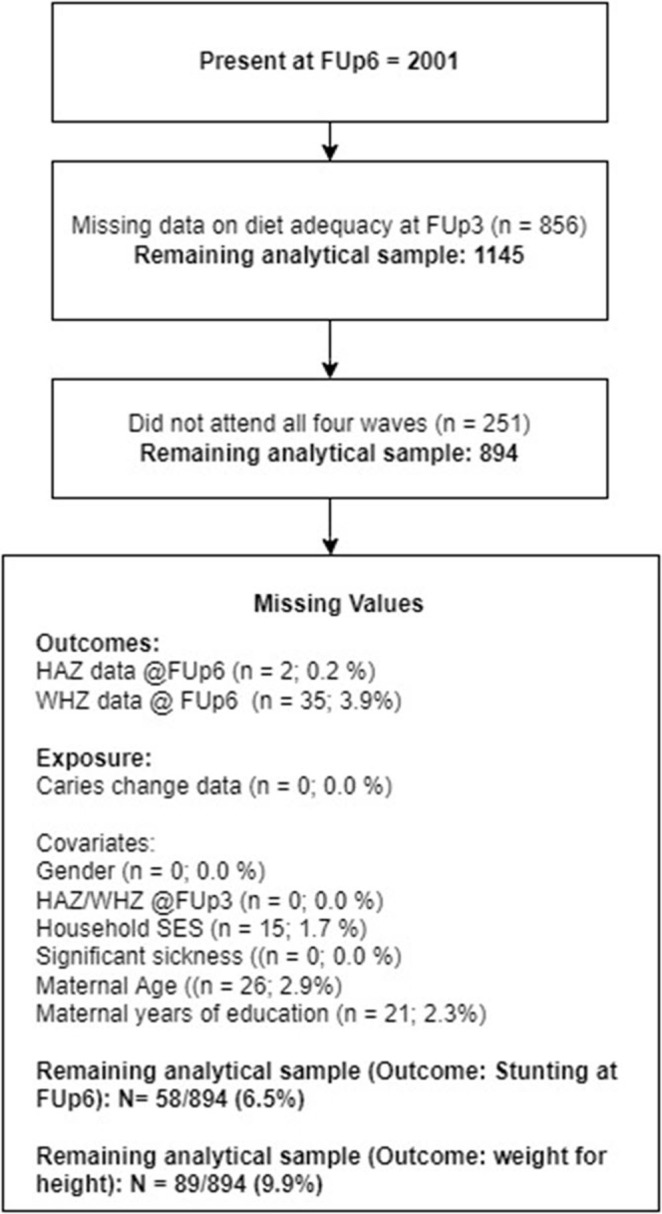
Flow chart demonstrating the selection of the analytical sample SES – socio-economic status; HAZ – height-for-age Z-score; WHZ – weight-for-height Z-score; FUp – follow-up.

The current analysis used a subsample of data from FUP3 through to FUP6 to maintain the temporal sequence between exposure and the outcome of faltering linear growth (stunting) or Ponderal growth. FUP6 was the most recent data available for analysis at the conceptualization of the study. Only individuals who were complementary feeding at FUP3 were included in the analysis. **Figure 2** presents an overview of the analytical sample.

### Statistical analysis

After the WHZ and HAZ classes were developed, the associations between the classes (WHZ and HAZ classes as outcome) were examined. Class groupings were used in two ways; first, to descriptively examine within class groups, the trajectories of children with and without caries. Second, associations between caries status and class group were tested by selecting the most favourable class as the reference group. Spearman’s Rho analyses were used to test the correlation between class grouping and exposure to ECC using the reference class group as the comparison. Exposure to cavitated caries lesions and pulpally involved teeth considered in separate models. In LCA, proper class assignment is not guaranteed because class assignment is based on probabilities, the exact number or percentage of sample members within each class cannot be determined [17]. Nevertheless, participants were classified in two ways: 1) participants were assigned to the trajectory class for which they had the highest posterior probability of membership; 2) weights, equal to the probability of membership of caries exposure from the LCA for each child, were used in the regression models. All analyses were carried out using the statistical software Stata (release 16; Stata Corporation, College Station, TX).

### Ethics

The protocol for the study was reviewed by the National Ethics Committee for Health Research, Ministry of Health Cambodia (117/NECHR) and the protocol for the secondary data analysis was reviewed by the Research Committee at the University of Puthisastra.

## RESULTS

**Table 1** presents data on the characteristics of the study cohort at FUP3, which for the purposes of this analysis will henceforth be referred to as Baseline. Children were on average 19 months of age, one in three had cavitated caries lesions, and one in twenty had one or more pulpally involved carious lesions. One in ten had ‘wasting’ at baseline and four in ten had ‘stunting’ at baseline.

**Table 1:** Baseline characteristics of the study cohort*

Co-variates	N or Mean	% or SD	Min-max
Female Gender. % (n/N)	462	51.7%	
Child Age (months). Mean (SD); min-max	20.0	SD 9.1	5.0, 41.3
Weight for Height z-score. Mean (SD); min-max	-0.94	SD 0.95	-5.19, 3.29
WHZ<-2	105	11.7%	
Height for Age z-score. Mean (SD); min-max	-1.65	SD 1.05	-5.74, 1.81
HAZ<-2	350	39.1%	
Mother’s age. Mean (SD); min-max	28.3	SD 6.2	16.2, 46.6
Mother’s education years. Mean (SD); min-max	5.4	SD 3.2	0.0, 17.0
Socio-economic index. % (n/N):^†^			
Lowest	204	23.2%	
Low	234	26.3%	
Medium	159	18.1%	
High	205	23.3%	
Highest	80	9.1%	
Has one or more Cavities. % (n/N)	270	30.2%	
Pulp involvement. % (n/N)	55	6.2%	

SD – standard deviation, WHZ – weight for height Z-score, HAZ – height for age Z-score

*Cohort N = 894.

^†^Data missing for 15 cases.

Figure S1 in the **Online Supplementary Document** presents descriptive WHZ growth trajectory data on each of the 5 classes showing within-class differences in growth trajectories by the presence of dental cavities. Children who had one or more cavities at FUP4 had lower WHZ scores across all three follow-up time points and across all 5-class groupings. Exposure to caries and pulpally involved teeth were positively correlated with membership in less favourable WHZ class groupings (Spearman’s Rho).

Figure S2 in the **Online Supplementary Document** presents descriptive HAZ growth trajectory data on each of the 6 classes showing within-class differences in growth trajectories by the presence of dental cavities at FUP4. Each of the trajectories appear to demonstrate consistent HAZ scores across the period of observation. Further, most children exhibit negative HAZ scores across all class groupings and time points.

Table S1 in the **Online Supplementary Document** presents data on the baseline characteristics of participants by the highest probability WHZ class membership. When considering caries experience, there was no difference among groups. Those in the Consistently Positive WHZ group and the Stable Normal WHZ group had more favourable SES profiles with lower proportions of children in the ‘lowest’ and ‘low’ strata. If the At Risk of Severe Acute Malnutrition group were to be excluded as being outliers, then there is a trend whereby the more favourable WHZ class groups included children who had a lower prevalence of carious teeth with pulpal involvement; Consistently Positive WHZ group = 3.6%; Stable Normal WHZ group = 4.2%. Less favourable WHZ groups were as follows; Increasing Risk of Wasting group = 6.5%; and Consistently Skinny group = 7.1%. Spearman’s Rho values affirmed that membership in less favourable groupings was correlated with the presence of pulpally involved teeth.

Table S2 in the **Online Supplementary Document** presents data on the baseline characteristics of participants by HAZ class membership. While the differences in sociodemographic and clinical characteristics by group membership were not present, there was a trend by which those children who were in the Consistently Tall group or who were in the Stable Normal HAZ group had a more favourable SES profile with higher proportions of children in the favourable SES strata. Children who were in the ‘Stable Normal HAZ’ group had the most severe caries experience. Exposure to caries and pulpally involved teeth were negatively correlated with membership in less favourable HAZ class groupings (Spearman’s Rho).

**Table 2** presents regression models for the likelihood of WHZ class membership by ECC status (presence of any cavities or presence of any carious teeth with pulpal involvement at FUP4). Having dental cavities or having pulpally involved teeth were associated with lower odds of being in a class group where the WHZ trajectory was favourable. Those with one or more carious teeth with pulpal involvement had higher odds of belonging to the less favourable WHZ class than those with cavities only. Those with one or more cavities or with one or more carious teeth with pulpal involvement had lower odds of belonging to the Consistently Positive WHZ group than the reference group. The E-values for the effect estimates were large making it unlikely that unmeasured confounding could negate the effects observed in the model (Table S3 in the **Online Supplementary Document**).

**Table 2:** Adjusted associations between WHZ trajectories and ECC Exposure compared with the reference class*

	Persistently at Risk of Severe Acute Malnutrition OR (95% CI)	Increasing Risk of Wasting OR (95% CI)	Consistently skinny OR (95% CI)	Stable Normal WHZ† OR (95% CI)	Consistently Positive WHZ OR (95% CI)
Cavities‡	1.31 (0.54-3.16)	1.16 (0.8-1.7)	1.23 (0.88-1.73)	1.0 (n/a)	0.27 (0.08-9.8)
Pulpal involvement§	3.11 (0.66-14.68)	2.06 (0.92-4.60)	2.01 (0.97-4.15)	1.0 (n/a)	0.06 (0.02-0.39)

WHZ – weight for height Z-score, OR – odds ratio, CI – confidence interval

*Adjusted multinomial odds ratio: gender, mother’s age, mother’s education, and socioeconomic status.

†Reference category.

‡Child is exposed to one or more cavities extending to the dentine during the first 1000 days of life.

§Child is exposed to a caries lesion extending to the pulp during the first 1000 days of life.

**Table 3** presents regression models for likelihood of HAZ class membership by disease exposure. There are consistently lower odds of participants with caries exposure (cavities, with or without pulpal involvement) being in a less favourable HAZ class grouping. The confidence intervals around each of the odds ratios are broad and suggest a large degree of uncertainty. This was also observed in the E-values (Table S4).

**Table 3:** Adjusted associations between HAZ trajectories and ECC exposure compared with the reference class*

	Persistent severe stunting OR (95% CI)	Consistently stunted OR (95% CI)	Consistently Borderline OR (95% CI)	Persistently short OR (95% CI)	Stable Normal HAZ† OR (95% CI)	Consistently tall OR (95% CI)
Cavities ‡	0.80 (0.34-1.92)	0.68 (0.40-1.17)	0.82 (0.53-1.29)	0.88 (0.56-1.38)	1.0 (n/a)	0.62 (0.24-1.61)
Pulpal involvement §	0.68 (0.08-5.84)	0.61 (0.19-1.93)	1.10 (0.47-2.56)	0.86 (0.36-2.07)	1.0 (n/a)	0.36 (0.05-2.85)

ECC – early childhood caries, HAZ – height for age Z-score, OR – odds ratio, CI – confidence interval

*Adjusted multinomial odds ratio: gender, mother’s age, mother’s education, and socioeconomic status.

†Reference category.

‡Child is exposed to one or more cavities extending to the dentine during the first 1000 days of life.

§Child is exposed to a caries lesion extending to the pulp during the first 1000 days of life.

## DISCUSSION

In this analysis, LCA methodology was used to examine the relationship trends between ponderal and linear growth and ECC. WHZ and HAZ trajectory class groups were used as an outcome measure which was more sensitive to the impact of chronic conditions than standard outcome measures such as stunting or wasting. This was especially important among the study population, which did not conform to the WHO normal population-level growth standards. An effect was observed whereby those who were exposed to ECC were more likely to belong to a more favourable HAZ class but there was a large degree of uncertainty around the effect. There was a clear association between exposure to caries and WHZ class membership whereby children with caries exposure were more likely to belong to WHZ classes with lower mean Z-scores over time. The study offers evidence that affirms the association between ECC and low WHZ scores.

Before considering the findings of the study in more detail it is appropriate to first examine the limitations of the study. The key limitations in the study are related to the sampling frame and that, after the exposure measure was taken, only 3 waves of data were available to characterize class membership. Having only 3-data points for characterizing class membership means that there would have been some loss of accuracy in determining differences among trajectories classes. The fact that the present sample characteristics are not generalizable to the broader population in Cambodia, creates an external validity problem. Children in the study sample are considered poor within the context of a Low-to-Middle income country given their province of origin in the northeast, least economically productive region of Cambodia. This economic disparity may drive the burden of stunting and wasting among the group and may mask some of the ECC-related impacts on growth.

There was also an issue of lower than desired statistical power due to low numbers among some HAZ and WHZ class subgroupings. Once class subgroupings are generated then the number of children classified as having an exposure, particularly an exposure to a carious tooth with pulpal involvement, is low and that contributes to a lack of precision in the findings.

Children who were exposed to caries lesions in their first 1000 days of life were more likely to belong to WHZ classes with lower Z-scores. There was an expected trend where exposure to more severe carious lesions with pulpal involvement, as opposed to cavities extending to dentine only, were at a further increased risk of belonging to less favourable WHZ class. Simulation modelling could be a useful next step for estimating the potential benefit of a caries prevention and treatment interventions to reduce the risk of wasting malnutrition.

Ideally, a dynamic measure of caries exposure such as caries increment might be used; however, a decision made to prioritize the temporal relationship between exposure and trajectory class outcome and caries increment as an exposure would have compromised that relationship in the present data set. Caries lesions, particularly those where the pulp has become involved, lead to pain, loss of biting and chewing function, loss of sleep and an inflammatory response [5].

The data suggest that there is some effect whereby those children who are exposed to caries lesions have a higher chance of belong to a more favourable HAZ class grouping. The results suggest a high degree of uncertainty, specifically, broad confidence intervals, and small E-values relative to Odds Ratios. This suggests the possibility that there may be some unmeasured confounding in the modelling. One unmeasured covariate that could create such uncertainty is consumption of non-nutritious foods; in this sub-population, children in higher SES strata consume a greater quantity of Sugary sweetened beverages and packaged snacks, which drives a more severe manifestation of caries. This would mean that SES is both protective against stunting malnutrition and associated with a more severe caries experience. Given this, it is not possible to make any clear statements about the presence or directionality of any correlations between ECC and linear growth.

This study was limited to observing linear growth over a period of around 2 yearss, among children exposed to dental caries in their first 1000 days of life and among children who live in some of the poorest regions of Cambodia. It is of note that the mean trajectories among the different classes were flat, that within classes, there was little variation across the three time points. This could be a feature of children in their first few years of life where variation in linear growth could largely be accounted for by a mothers’ nutrition status, and in-utero health in addition to post-natal nutrition [18,19]. The classes with more favourable linear growth Z-scores featured children with more favourable sociodemographic profiles who may be likely to have access to better prenatal and postnatal diet/nutrition.

The findings of this LCA analysis are in contrast the findings from the Fit for School project. In that study, it was reported that 8- to 10 year-old children with more severe caries experienced a greater prevalence of stunting malnutrition [20]. The contrasting conclusions between the present and previous analysis could partly be explained by the timing of exposure (caries), stage of dental development, and the lag period for development of stunting in children. Given the biological plausibility for a negative relationship between untreated caries lesions and stunting malnutrition, mediated through pain, inflammation, and difficulty eating or sleeping, it seems less plausible that caries might have a positive effect on linear growth. Such a relationship is more likely to be explained by confounding factors. In addition, larger studies with long-term follow up period (into late childhood) are needed to be able to draw conclusions on the relationship between exposure to caries lesions and HAZ scores.

Dental caries is a preventable and treatable disease and interventions to prevent ECC have been demonstrated to be effective, and are justifiable on their own as a means of reducing pain and suffering among children in high caries populations [21]. The results of this analysis suggest that reducing the prevalence and severity of ECC could also have a positive effect on child growth and development. Therefore, interventions aiming to address the UN Sustainable Development Goals 2030 (SDG), specifically SDG 3 ‘Good Health and Well-being’, should include oral health components. Given the general lack of funding for oral health interventions, integrating oral health programing with those supporting normal child growth and development in the early years would be an appropriate way to reduce the impacts of oral disease. Such an approach is supported by WHO [22,23].

In the present study only a minimally and guaranteed set of confounding factors were included. Baseline nutrition status was not included as covariates in order to avoid over adjustment particularly given that the outcomes were measured at multiple time points [24]. Also, variables such as water and sanitation as well as dietary adequacy, previously published as being associated with wasting malnutrition were not included in the present model [8,9,25,26]. This is not thought to influence the directionality and size of the effect, as the E-values suggest that unmeasured confounding would have to be large relative to the observed effects. Further, unmeasured confounding would need to be greater than that reported in previous investigations from the same cohort [8,27]. The implications are that this initial descriptive exercise should be followed up by a more intensive research study using long-term cohorts specifically designed to explore the relationship between ECC and growth development.

## CONCLUSIONS

Children with ECC in their first 1000 days of life were more likely to belong to unfavourable WHZ trajectory class groupings. The present analysis added evidence to the hypothesis that ECC may be a modifiable risk factor for unfavourable linear and ponderal growth.

## Additional material


Online Suplementary Document
