# Supplementary material for: Associations between dental caries and ponderal growth in children: A Cambodian study
Source: J Glob Health. 2022 Jun 18;12:04046. doi: 10.7189/jogh.12.04046 (PMC9204672; doi:10.7189/jogh.12.04046)
Supplement: Online Suplementary Document [file jogh-12-04046-s001.pdf]

Supplementary Material

Table S1. Baseline characteristics of the study cohort by WHZ class groupings determined by highest probability

	Persistently at Risk of Severe Actue Malnurtition N = 22 (2.5%)		Increasing Risk of Wasting N = 230 (25.7%)		Consistently Skinny N = 378 (42.3%)		Stable Normal WHZ * N = 236 (26.4%)		Consistently Positive WHZ N = 28 (3.1%)	
Characteristic	N/Mean	%/SD	N/Mean	%/SD	N/Mean	%/SD	N/Mean	%/SD	N/Mean	%/SD
Female Gender	14	63.6%	111	48.3%	194	51.3%	131	55.5%	12	42.9%
Age (months)[c]	13.8	SD 7.1	19.7	SD 9.2	21.3	SD 9.2	19.2	SD 9.6	17.5	SD 9.5
WHZ score at FUP3[†]	-2.59	SD 0.65	-1.68	SD 0.64	-1.01	SD 0.69	-0.13	SD 0.61	0.61	SD 1.08
HAZ score at FUP3[†]	-2.50	SD 1.13	-1.92	SD 0.98	-1.76	SD 0.96	-1.21	SD 1.02	-0.81	SD 1.26
Mother's age (years)	28.4	SD 8.7	28.2	SD 5.8	28.3	SD 6.2	28.3	SD 6.5	30.1	SD 5.7
Mother's education years	4.4	SD 2.8	5.3	SD 3.3	5.2	SD 3.1	5.8	SD 3.2	6.6	SD 4.3
Socioeconomic index										
Lowest	4	18.2%	53	23.5%	91	24.4%	53	22.9%	3	11.1%
Low	8	36.4%	63	27.9%	99	26.5%	55	23.8%	6	22.2%
Medium	3	13.6%	41	18.1%	66	17.7%	41	18.2%	7	25.9%
High	6	27.3%	51	22.6%	81	21.7%	61	26.4%	6	22.2%
Highest	1	4.5%	18	8.0%	36	9.7%	20	8.7%	5	18.5%
One or more Cavities at FUP4	7	31.8%	68	29.6%	119	31.5%	69	29.2%	7	25.0%
Spearman's Rho.	0.016		0.004		0.024		Reference category		-0.029	
Implied power	83.0%		73.3%		98.5%		Reference category		98.8%	
One or more teeth with Pulp involvement at FUP4	2	9.1%	15	6.5%	27	7.1%	10	4.2%	1	3.6%
Spearman's Rho.	0.064		0.051		0.059		Reference category		-0.010	
Implied power	>99.9%		99.1%		99.8%		Reference category		84.8%	

Abbreviations as follows, WHZ = Weight for Height Z-scores; HAZ = Height for Age Z-score; FUP = Follow-up; SD = Standard Deviation.

*Stable Normal WHZ group is the reference class grouping for WHZ models

[†]P-value = <0.001; t-test for difference in characteristic by WAZ class membership

Table S2. Baseline characteristics of the study cohort by HAZ class groupings determined by highest probability

Characteristic	Persistently Severely Stunted N = 31 (3.5%)		Consistently Stunted N = 154 (17.2%)		Consistently Borderline N = 312 (34.9%)		Persistently Short N = 245 (27.4%)		Stable Normal HAZ[b] N = 122 (13.7%)		Consistently Tall N = 30 (3.4%)	
	N/Mean	%/SD	N/Mean	%/SD	N/Mean	%/SD	N/Mean	%/SD	N/Mean	%/SD	N/Mean	%/SD
Female Gender	13	41.9%	85	55.2%	163	52.2%	133	54.3%	54	44.3%	14	46.7%
Age (months)	18.1	SD 9.2	20.1	SD 8.9	20.1	SD 9.4	20.1	SD 9.3	20.7	SD 8.5	18.1	SD 9.2
WHZ -score	-1.27	SD 1.02	-1.31	SD 0.96	-0.97	SD 0.91	-0.88	SD 0.85	-0.59	SD 0.94	-0.19	SD 1.18
Height for Age z-score	-3.68	SD 0.76	-2.71	SD 0.47	-1.94	SD 0.57	-1.22	SD 0.58	0.44	SD 0.59	0.58	SD 0.59
Mother's age (years)	26.1	SD 6.4	27.2	SD 6.2	28.7	SD 6.4	28.3	SD 5.7	29.0	SD 6.4	30.1	SD 6.7
Mother's education years	3.1	SD 2.6	4.1	SD 3.1	5.7	SD 3.0	5.5	SD 3.1	5.9	SD 3.3	7.8	SD 4.2
Socioeconomic index												
Lowest	11	35.5%	55	36.2%	61	20.2%	53	21.8%	21	17.4%	3	10.0%
Low	8	25.8%	32	21.1%	87	28.8%	66	27.2%	31	25.6%	7	23.3%
Medium	5	16.1%	25	16.4%	65	21.5%	38	15.6%	21	17.4%	5	16.7%
High	5	16.1%	28	18.4%	63	20.9%	65	26.7%	35	28.9%	9	30.0%
Highest	2	6.5%	12	7.9%	26	8.6%	21	8.6%	13	10.7%	6	20.0%
One or more Cavities at FUP4	8	25.8%	35	22.7%	92	29.5%	79	32.2%	46	37.7%	10	33.3%
Spearman's Rho	-0.100		-.163		-.079		-0.054		Reference category		-0.036	
Implied power	>99.9%		>99.9%		>99.9%		>99.9%		Reference category		96.6%	
One or more teeth with Pulp involvement at FUP4	1	3.2%	5	3.2%	21	6.7%	17	6.9%	10	8.2%	1	3.3%
Spearman's Rho	-0.077		-.108		-0.026		-0.023		Reference category		-0.075	
Implied Power	>99.9%		>99.9%		99.8%		99.1%		Reference category		99.9%	

[a] Abbreviations as follows, WHZ = Weight for Height Z-scores; HAZ = Height for Age Z-score; FUP = Follow-up; SD = Standard Deviation.

[b] Reference class for HAZ modelling

Table S3 – E-values for relationship between ECC and WHZ trajectory

	Persistently at Risk of SAM vs Stable Normal WHZ		Increasing Risk of Wasting vs Stable Normal WHZ		Consistently Skinny vs Stable Normal WHZ		Consistently Positive WHZ vs Stable Normal WHZ	
	**E-Value**	**E-Value for CI**	**E-Value**	**E-Value for CI**	**E-Value**	**E-Value for CI**	**E-Value**	**E-Value for CI**
Cavities*	1.95	3.11	1.59	1.81	1.76	1.53	24.49	6.87
Pulp involvement†	5.67	2.40	3.54	1.39	3.43	1.21	32.83	4.57

Abbreviations; ECC = Early Childhood Caries; WHZ = Weight for Height Z-score; CI = Confidence Interval

*Child is exposed to one or more cavities extending to the dentine during the first 1000 days of life;

†Child is exposed to a caries lesion extending to the pulp during the first 1000 days of life

Table S4 – E-values for relationship between ECC and HAZ trajectory [a]

	Persistent Severe Stunting vs Stable Normal HAZ		Consistently Stunted Vs Stable Normal HAZ		Consistently Borderline Vs Stable Normal HAZ		Persistently Short Vs Stable Normal HAZ		Consistently Tall vs Stable Normal HAZ	
	**E-value**	**E-value for CI**	**E-value**	**E-value for CI**	**E-value**	**E-value for CI**	**E-value**	**E-value for CI**	**E-value**	**E-value for CI**
Cavities*	1.81	5.33	2.30	4.44	1.74	3.18	1.53	2.97	2.61	7.80
Pulp involvement†	2.30	24.49	2.66	10.00	1.43	3.68	1.60	5.00	5.00	39.49

Abbreviations; ECC = Early Childhood Caries; HAZ = Height for Age Z-score; CI = Confidence Interval

*Child is exposed to one or more cavities extending to the dentine during the first 1000 days of life;

†Child is exposed to a caries lesion extending to the pulp during the first 1000 days of life

Figure S1 – WHZ trajectory by class membership and caries exposure*

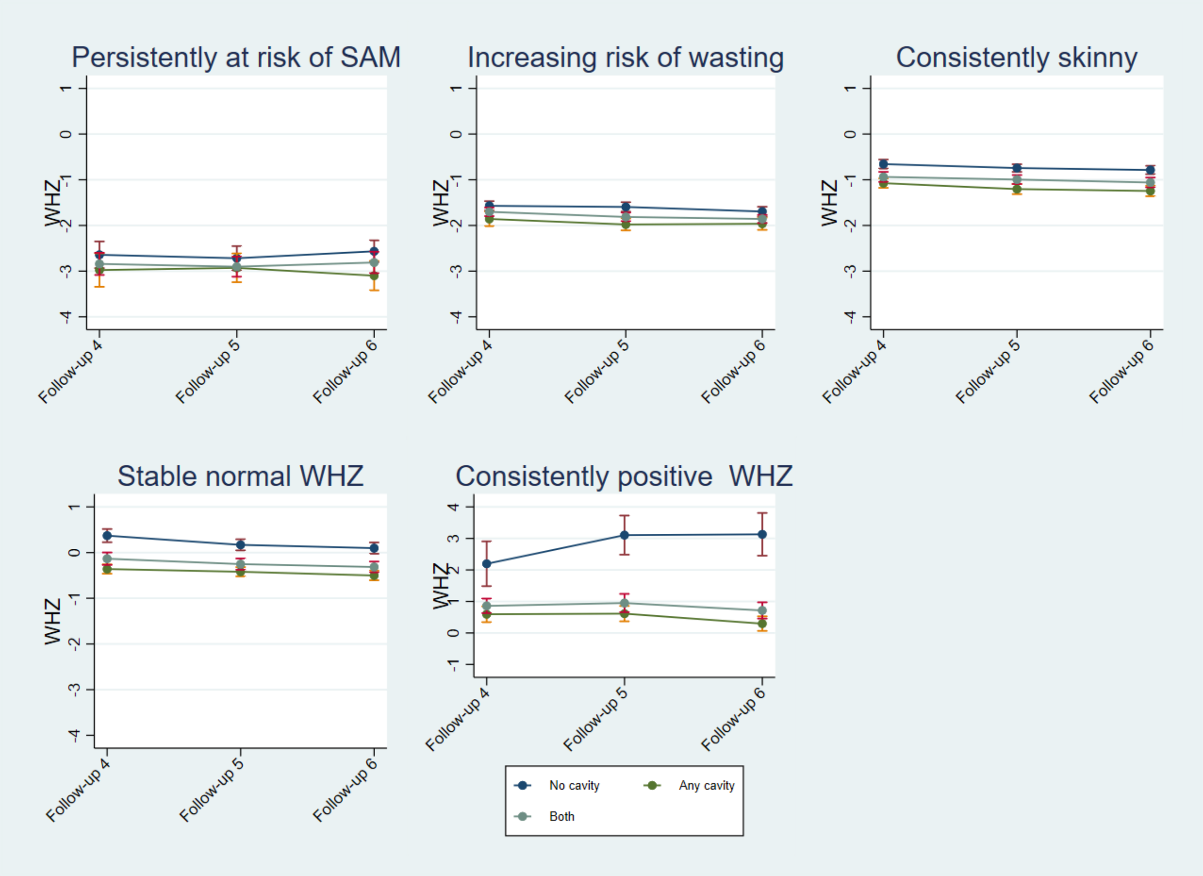

Abbreviations: WHZ = Weight for Height Z-score; SAM = Severe Acute Malnutrition;

*Definitions in legend; Both (grey line) refers to all children in the class grouping; No cavity (blue line) refers to those participants who did not have any cavitated dental caries lesions; Any cavity (green line) refers to the participants within the class group who had one or more cavitated caries lesions extending to dentine.

Figure S2 – HAZ trajectory by class membership and caries exposure*

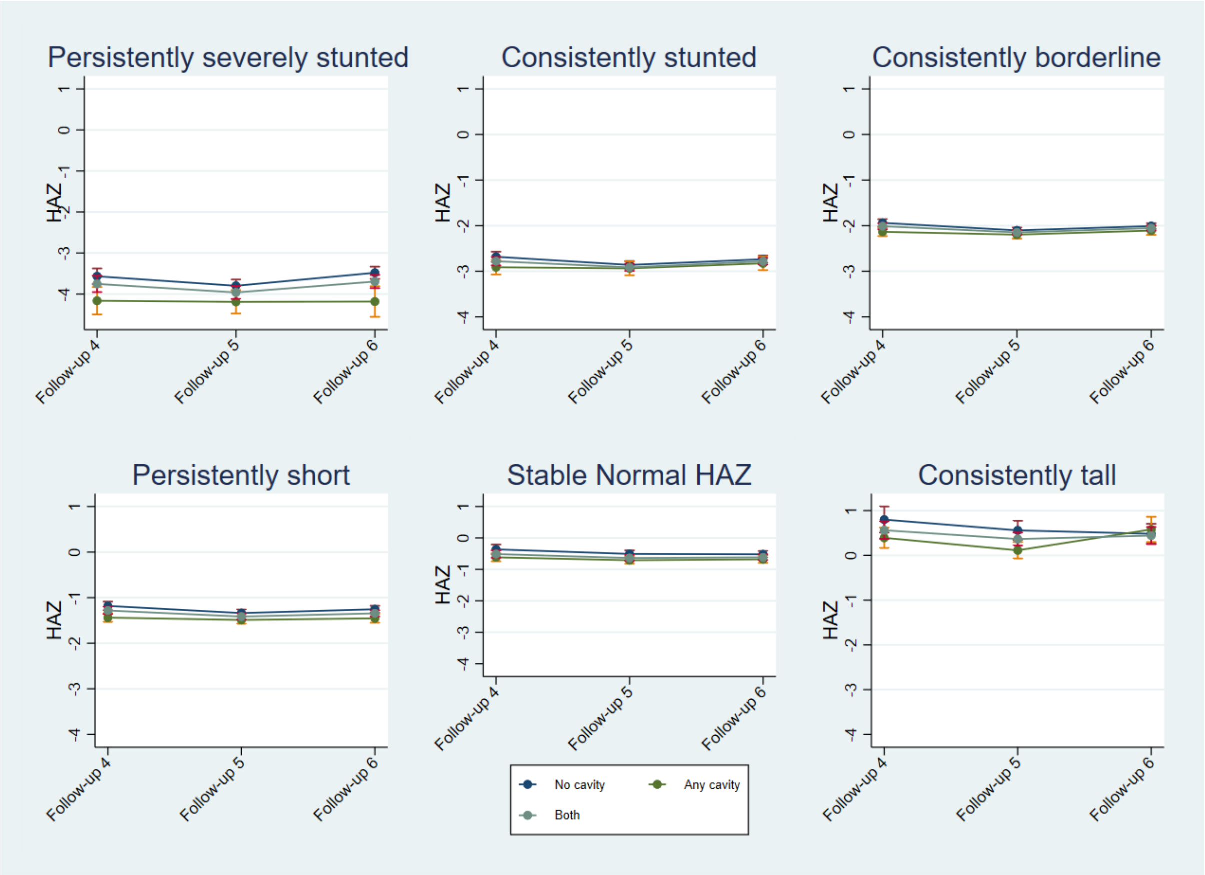

Abbreviations HAZ = Height for Age Z-score; SAM = Severe Acute Malnutrition;

*Definitions in legend; Both (grey line) refers to all children in the class grouping; No cavity (blue line) refers to those participants who did not have any cavitated dental caries lesions; Any cavity (green line) refers to the participants within the class group who had one or more cavitated caries lesions extending to dentine.
